# Thermoregulatory Stress as Potential Mediating Factor in the NTP Cell Phone Tumor Study

**DOI:** 10.1002/bem.22284

**Published:** 2020-07-21

**Authors:** Jens Kuhne, Janine‐Alison Schmidt, Dirk Geschwentner, Blanka Pophof, Gunde Ziegelberger

**Affiliations:** ^1^ Federal Office for Radiation Protection Neuherberg Germany

**Keywords:** hyperthermia, hypothermia, metabolism, SAR, thermal effect

## INTRODUCTION

To date, the National Toxicology Program (NTP) cellphone study is the most comprehensive animal experiment on the health effects of chronic radiofrequency electromagnetic radiation (RF‐EMR) exposure at high levels. Since the outcome of the study is considered to be of high relevance for human health, the final reports have attracted international attention. The strengths and weaknesses of the study have been reviewed by several scientists (e.g. Lin [2018]; Melnick [2019]), institutions (e.g. Australian Radiation Protection and Nuclear Safety Agency (ARPANSA) [2018]; Food and Drug Administration (FDA) [2018]; Bundesamt für Strahlenschutz (BfS) [2019]; Food and Drug Administration (FDA) [2020]), and organizations (e.g. Beratende Expertengruppe NIS (BERENIS) [2018]; International Commission on Non‐Ionizing Radiation Protection (ICNIRP) [2020]).

In the main NTP study, Sprague–Dawley (SD) rats and B6C3F1 mice of both sexes were exposed to GSM (TDMA) and IS‐95 (CDMA) RF‐EMR at 900 MHz (rats) and 1,900 MHz (mice) for 2 years. The unrestrained, individually housed animals were exposed in reverberation chambers that ensured a high time‐average spatial homogeneity of the exposure [Capstick et al., 2017; Gong et al., 2017]. During the 2‐year phase the animals were exposed to whole‐body specific absorption rates (wbSAR) of up to 6 W/kg (rats) and 10 W/kg (mice) in an intermittent 10‐min field on, 10‐min field off scheme with two long exposure breaks from 7 am to 11 am and from 2 pm to 3:40 pm each day, resulting in a daily net exposure duration of 9 h 10 min. The average wbSAR during field on times was kept constant throughout the whole life of the animals by adjusting the applied RF‐EMR power according to the body mass of the animals. The most prominent findings were observed in exposed male rats, including increased incidence of malignant schwannomas in the heart (GSM and IS‐95: statistically significant positive trend with increasing wbSAR; IS‐95: statistically significant increase in the 6 W/kg exposure group), reduced severity of chronic progressive nephropathy (CPN), and a number of symptoms that were judged to be correlated to this kidney disease, as well as a higher survival rate compared with sham‐exposed rats. In female rats, the number of lesions and severity of health effects was significantly lower compared with male rats. The smallest effect size was observed in mice of both sexes, although mice were exposed to nearly twice the wbSAR used in the rat studies. On the basis of the incidence of malignant schwannomas in the heart of RF‐EMR‐exposed male rats, the NTP concluded that under the whole‐body exposure conditions of the 2‐year study, there is clear evidence (highest evidence level in NTP classification) of carcinogenic activity of GSM (TDMA) and IS‐95 (CDMA) RF‐EMR in male rats. For a detailed description of the study design and findings, the reader is referred to the original technical reports [National Toxicology Program (NTP), 2018a,b].

**Figure 1:**
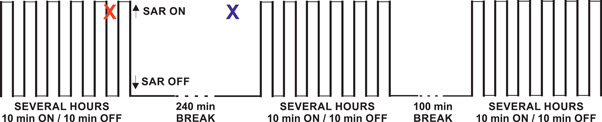
Schematic representation of the temperature measurements during the daily whole‐body specific absorption rates pattern in Wyde et al. [2018]. Body temperatures were measured via interscapular subcutaneously implanted microchips and readers within 1–2 min after the end of a 10‐min field‐on period (indicated by red X) as well as 3 h after initiation of the long 4‐h exposure break (indicated by blue X).

In the main 2‐year study, body temperature measurements were not recorded. In order to select appropriate wbSAR levels for the main study, temperature measurements were carried out in pilot studies [Wyde et al., 2018]. In these pilot studies, rats and mice of different sex and age were exposed to GSM and IS‐95 RF‐EMR at wbSAR levels of up to 12 W/kg in the same intermittent scheme as applied in the main study. Body temperatures were measured individually for each rat and mouse via interscapular subcutaneously implanted microchips and readers shortly (within 1–2 min acc. to the authors) after the end of a 10‐min field‐on period at nine time points (distributed over the 5‐day study duration), as well as 3 h after initiation of the 4‐h exposure break (see Fig. 1). A wbSAR of 6 W/kg was the highest exposure level that did not lead to average subcutaneous (SC) body temperature elevations of more than 1°C in rats and was therefore selected as maximal exposure level during the 2‐year chronic exposure rat study. However, the following analysis of the published data suggests that the average values of the temperature increase at 6 W/kg can only be regarded as lower bounds of the maximum (exposure pattern‐related) temperature fluctuation (compared with sham‐exposed) of the 2‐year study.

## MATERIALS AND METHODS

Published SC body temperature data listed in the supplementary Tables ST1–ST14 (day 1–day 5) of Wyde et al. [2018] were analyzed using MATLAB (The MathWorks, Natick, MA). SC body temperatures $T_{i}$, standard deviations $\sigma_{i}$, and number of animals per datapoint $n_{i}$ for the measurements at different time points after first initiation of exposure of the 6 W/kg and sham‐exposed (0 W/kg) subgroups of GSM/IS‐95, male/female, young/aged, mice/rats were extracted from the tables. Data with missing exposure or sham‐exposed counterpart were omitted. From the k data points of each subgroup, the total number of measurements $N=\sum_{i=1}^{k}n_{i}$ and the average SC body temperature $\bar{T}=\frac{1}{N}\times \sum_{i=1}^{k}n_{i}\times T_{i}$ were calculated. The variance $\sigma^{2}$ of each subgroup was calculated using Equation (1).

(1) $$\sigma^{2}=\frac{1}{N-1}\times \left(\sum_{i=1}^{k}n_{i}\times {\sigma_{i}}^{2}+\sum_{i=1}^{k}n_{i}\times {{(T}_{i}-\bar{T})}^{2}\right)$$

Pairwise exposure‐induced temperature deviations ${∆T}_{i}$ were calculated using Equation (2) by subtracting the data of the time‐matched 0 W/kg subgroup $T_{i_{c}}$ from the respective 6 W/kg subgroup $T_{i_{e}}$.

(2) $${∆T}_{i}=T_{i_{e}}-T_{i_{c}}$$

The subgroup average exposure‐induced temperature deviation $∆\overset{-}{T}$ was calculated in the same fashion using the average SC body temperatures of the 0 and 6 W/kg subgroups ${\bar{T}}_{c}$ and ${\bar{T}}_{e}$ to account for potentially different animal numbers that relate to each datapoint.

(3) $$∆\bar{T}={\bar{T}}_{e}-{\bar{T}}_{c}$$

The corresponding standard error of the mean temperature difference ${\text{SEM}}_{∆}$ was calculated using Equation (4) by taking possible correlations in the 0 and 6 W/kg datasets due to potentially similar diurnal variations in the animals into account:

(4) $$\begin{array}{l} {\text{SEM}}_{∆}=\frac{1}{k}\\ \;\times \sqrt{\begin{array}{l} \sum_{i=1}^{k}\frac{1}{n_{i_{e}}}\times \sigma_{e}^{2}+\sum_{i=1}^{k}\frac{1}{n_{i_{c}}}\times \sigma_{c}^{2}\\ -2\times \sum_{i=1}^{k}\frac{1}{\max\left(n_{i_{e}},n_{i_{c}}\right)}\times \text{cov}(T_{e},T_{c}) \end{array}} \end{array}$$

Here, $\sigma_{e}^{2}$ and $\sigma_{c}^{2}$ denote the variances of the temperature data of the 0 and 6 W/kg subgroups, respectively. GSM and IS‐95 data of the subgroups were merged into five animal groups with the parameters listed in Table 1, and $\bar{∆T}$ and ${\text{SEM}}_{∆}$ were calculated as described in Equations (3) and (4). Data of temperature measurements shortly after exposure cessation and temperature measurements after the long exposure breaks were treated separately.

**Table 1:** Parameters of the Animal Group Data

Animal group	Body mass interval (g)	Total number of temperature measurements shortly after exposure cessation (exposed/sham‐exposed); Anderson–Darling normality tests for normality of ${∆T}_{i}$	Total number of temperature measurements after long exposure break (exposed/sham‐exposed); Anderson–Darling normality tests for normality of ${∆T}_{i}$
Young mice (M + F)	18–22	180/179; *P* > 0.05	40/40; *P* > 0.05
Aged mice (M + F)	50–57	178/177; *P* > 0.05	40/40; *P* > 0.05
Young rats (M + F)	120–158	135/135; *P* > 0.05	30/30; *P* > 0.05
Aged rats (F)	261–298	84/85; *P* > 0.05	20/20; *P* > 0.05
Aged rats (M)	470–504	85/84; *P* > 0.05	20/20; *P* > 0.05

## RESULTS

**Figure 2:**
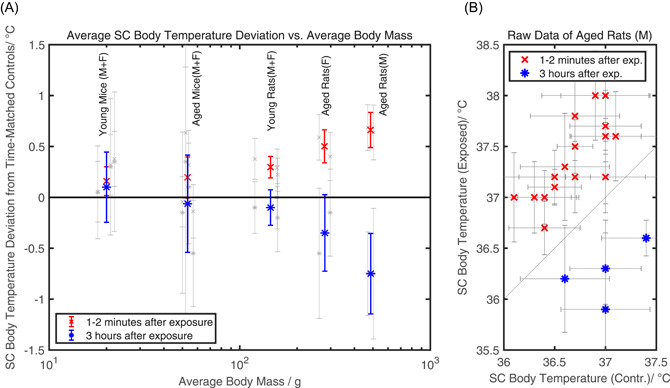
Subcutaneous (SC) body temperature measurements of the 6 W/kg exposure groups. (**A**) Compilation of the SC body temperature data given in the supplementary tables of Wyde et al. [2018]. The gray data points represent the exposure pattern‐induced average SC body temperature deviation from sham‐exposed (control) for each 6 W/kg exposure group (GSM/IS‐95, male/female, young/aged, mice/rats). The SC body temperature elevation measured 1–2 min after exposure cessation was averaged for exposure groups with similar average body mass (red data points, see MM section and Table 1 for additional information on grouping). The same procedure was performed for the data of the long exposure break‐induced average SC body temperature reduction measured 3 h after exposure cessation (blue data points). The error bars represent the respective 95% confidence Intervals ($\bar{∆T}$ ± 1.96 × ${\mathrm{SEM}}_{∆}$). A clear body mass to body temperature elevation relationship is observed with higher body mass associated with more pronounced exposure pattern‐induced temperature fluctuations. (**B**) Raw temperature data of 6 W/kg GSM radiofrequency electromagnetic radiation (RF‐EMR) exposed, IS‐95 RF‐EMR exposed, and time‐matched sham‐exposed aged male rats. All raw data points of the measurement 1–2 min after exposure cessation (red) indicate SC body temperature elevation, and all raw data points of the measurement during the long exposure break (blue) indicate SC body temperature reduction. Both measurements are positively correlated with the body temperature of the time‐matched sham‐exposed animals (elevation: *c*_pearson_ = 0.69, reduction: *c*_pearson_ = 0.57).

A compilation of the SC body temperature data of the 0 and 6 W/kg subgroups of different species, sex, and age is presented in Figure 2. The magnitude of the exposure‐induced SC body temperature elevation measured shortly after several cycles of the intermittent 10‐min field on, 10‐min field off exposure is associated with the species‐ and age‐related average body mass (Fig. 2A, red data points). For aged male rats, a more pronounced exposure‐induced average SC body temperature elevation was measured ($\bar{∆T}$= 0.66 °C) than for animals with low body mass such as young mice ($\bar{∆T}$= 0.1 °C). It is important to note that the 1‐ to 2‐min time lag between end of exposure and temperature measurement introduces a systematic underestimation of the magnitude of the exposure‐induced SC body temperature elevation. SC body temperatures were also measured during the long exposure pauses 3 h after the last exposure (Fig. 2A, blue data points). Interestingly, the SC body temperatures of some exposed groups were lower than the ones of the respective sham‐exposed groups. This exposure break‐induced average SC body temperature decrease exhibits a strong body mass dependency as well. No difference is visible in mice, whereas the average SC body temperature of exposed aged male rats was 0.75°C lower than the sham‐exposed group. The exposure pattern‐induced SC body temperature fluctuation (maximum elevation minus maximum reduction) measured in the pilot study, therefore, adds up to a lower bound of approximately 1.4°C for aged male rats with an average body mass of 487 g.

A closer analysis of the raw SC body temperature data of aged male rats reveals that these exposure pattern‐induced fluctuations at least partly add to the natural daily temperature fluctuations (Fig. 2B): The overall temperature distribution of the 6 W/kg exposed group (red + blue along the *y*‐axis) is substantially broader than the overall temperature distribution of the time‐matched sham‐exposed group (red + blue along the *x*‐axis), which is an estimate of the natural daily fluctuation. Additionally, the SC body temperature elevation data of exposed and time‐matched sham‐exposed animals are positively correlated (red data points: *c*_pearson_ = 0.69) indicating that the exposure‐induced temperature elevation is independent from the actual temperature of the animal without exposure. For the SC body temperature decrease, it is unclear whether the positive correlation (*c*_pearson_ = 0.57) is a true effect because only few measurement points are available.

## DISCUSSION

### RF‐EMR Exposure Affects Metabolic Activity

In the NTP study, rats and mice were kept at ambient temperatures of approximately 22°C, which is approximately 6–8°C below their thermoneutral zone [Gordon, 1990, 2012]. Therefore, a significant amount of heat has to be generated by metabolic activity in order to maintain body temperature and due to their high body surface to mass ratio (SMR), metabolic activity is higher in small animals compared with heavier ones [Gordon and Ferguson, 1984; Gordon, 1990]. In the ambient temperature range used in the NTP study, the resting metabolic rate of mice is expected to be twice as high as that of rats, although this is affected by several factors such as type of cage bedding, air movement within the cage, genetic strain, gender, and age of the animals. Calorimetry data collected from individually housed mice and rats show that, at 22°C, mice generate approximately 20 W/kg [Gordon, 2012], whereas for SD rats with body mass of 600–800 g a value of 6 W/kg can be estimated from the literature (9‐month‐old rat with special diet) [Ghibaudi et al., 2002]. It is important to note that basal metabolic rate of rodents generally declines with age [Balmagiya and Rozovski, 1983; Gordon, 1993]. Under cool conditions where metabolic rate is elevated above the basal levels that would be observed at thermoneutrality, the energy from RF‐EMR is utilized in place of metabolic energy to thermoregulate in the cold [Gordon, 1987; Taberski et al., 2014]. If ambient temperature is within the thermoneutral zone or wbSAR is too high, the animal cannot lower metabolic rate below basal levels and the RF‐EMR energy leads to a marked heat stress. Animals with low body mass and high metabolic activity, like mice, have a higher capacity to compensate for the additional wbSAR‐dependent heat generation by lowering their own metabolism. Under the NTP housing conditions, the 6 W/kg exposure was likely beneficial for the thermal comfort of mice, and it is thus not surprising that no increase in SC body temperatures of mice was observed.

Besides the reduction of the metabolic activity in rats exposed to RF‐EMR at low ambient temperatures, heat‐acclimated rats show a decrease in both resting metabolic rate and food intake as well as an increase in water intake [Gordon, 1990]. Unfortunately, metabolic parameters have not been recorded in the NTP study. Due to considerations regarding energy balance, it is reasonable to expect that the metabolism of male rats has been significantly reduced upon whole‐body RF‐EMR absorption, which is also in line with the observed decrease of the SC body temperature during long exposure breaks. Some unexpected observations of the main 2‐year study further support this hypothesis: The NTP scientists explained the prolonged survival of exposed male rats by the significantly reduced severity of CPN and corresponding side effects. Heat‐induced changes in metabolism might be the explanation for the higher survival since a reduced metabolic activity leading to lower food intake is in line with the observations regarding CPN: “A number of factors, mainly dietary manipulations, have been shown to modify the expression of CPN. Amongst these, restriction of caloric intake is the most effective for inhibiting the disease process” [Hard and Khan, 2004]. An increase of the mean life expectancy and a reduction in incidence of chronic nephropathy caused by restriction of food intake (by time‐scheduled feeding) has also been reported earlier for another strain of SD rats [Deerberg et al., 1990].

### Evidence for Exposure Pattern‐Induced Thermoregulatory Stress That Increases with Study Duration

The data of the pilot studies indicate that the NTP exposure pattern leads to SC body temperature elevation during the cyclic intermittent exposure and SC body temperature reduction during the long exposure breaks in animals that exceed a body mass of approx. 100 g (Fig. 2A). Due to the subcutaneous interscapular location of the microchip, deviations of the recorded temperatures from the absolute body core temperatures cannot be ruled out. However, by comparing the SC body temperatures from exposed animals with SC body temperatures from time‐matched sham‐exposed animals as shown in Figure 2, such systematic errors can be reduced.

The observed SC body temperature elevation in rats during the cyclic intermittent exposure indicates hyperthermia, as it is in line with earlier investigations on unrestrained adult SD rats at ambient temperatures of 20°C that revealed a threshold wbSAR of 1.6 W/kg above which colonic temperature begins to rise with a slope of 0.6 °C/W/kg [Gordon et al., 1986]. From this data, a 1°C increase of body core temperature is expected in adult SD rats for a continuous wave wbSAR of 3.2 W/kg (approx. the average value of the 6 W/kg intermittent exposure).

The highly reproducible long‐lasting decrease in SC body temperature measured 3 h after switching off the exposure (Fig. 2B) points to a severe physiological change. A strong vasoconstriction below the hairy skin on the back of the rat (insulating the sensor from the core) could lead to the observed effect. However, as the ambient temperature is well below the lower critical temperature of normothermia, vasoconstriction can be expected in the sham‐exposed group as well. Therefore, it is possible that the SC body temperature decrease during the long breaks indicates hypothermia, a true reduction of the body core temperature. A decreased body core temperature (rectal measurements) shortly after cessation of RF‐EMR exposure has also been reported earlier in mice [Ebert et al., 2005]. However, we are not aware of rodent studies that reported an exposure cessation‐induced body core temperature decrease lasting for several hours.

The exposure pattern‐induced temperature fluctuations strongly depend on body mass and intensify as animals gain further mass. For the 6 W/kg exposure group of aged male rats with an average body mass of 487 g, the lower bound of the average exposure pattern‐induced SC body temperature fluctuation was approx. 1.4°C (elevation: 0.66°C, reduction: −0.75°C). There is evidence that for most of the main 2‐year study, the average SC body temperature fluctuation of male rats in the 6 W/kg exposure group was higher than reported in the pilot studies because during 77% of the study duration, the average body mass of male rats was higher than 487 g. As the body mass of individual animals is distributed around the average value (standard deviation approx. 10% of the mean), even higher fluctuations might have occurred for some individuals. Additionally, a further increase in body temperature fluctuation might have occurred because old SD rats have a reduced ability to regulate body temperature by tail vasodilation [Cox et al., 1981]. It is important to note that the exposure pattern‐induced fluctuations of SC body temperature can be considered as adding to the natural diurnal temperature variations of the animals, which indicates that the total (natural + exposure‐induced) temperature fluctuation of the animals exceeded normal physiological range (Fig. 2B).

In the main 2‐year study, a huge effort was taken to ensure a constant average wbSAR throughout the entire life of the rodents by adjusting power output to body size [Capstick et al., 2017; Gong et al., 2017]. This procedure might have biased the study outcome as the resulting body mass‐dependent thermal load was unevenly distributed among species and sex and increased during the study duration.

### Chronic Thermoregulatory Stress Might Have Led to Some of the Findings of the NTP Study

The study findings in heavier animals with expected higher body temperature fluctuations were more robust than the findings in less heavy animals. The strongest evidence for an adverse effect was found in the heart of male SD rats such as a statistically significant positive exposure‐related trend of malignant schwannoma incidence, statistically significant elevated incidence of cardiomyopathy in the right ventricle in the higher exposure groups, and exposure‐related increases in endocardial Schwann cell hyperplasia (not statistically significant). The effect size in male rats increased with increasing wbSAR, but no consistent effect by the type of modulation (TDMA or CDMA) has been found. At the time when the first malignant schwannoma was detected in the heart of a male rat (day 488, see table 47; National Toxicology Program (NTP) [2018a]), the exposure pattern‐induced temperature fluctuation had likely exceeded 1.4°C for more than 300 days (the average body mass of the rats was 30% higher than the maximum value used in Wyde et al. [2018]).

The heart of SD rats seems to be especially targeted upon forced body temperature fluctuation as it was shown that under certain circumstances, both heart rate and blood pressure increase significantly upon intense RF‐EMR body heating [Frei et al., 1989; Jauchem and Frei, 1992; Lin et al., 1994] as well as in rats that undergo hypothermia [Lin et al., 1994], and cardiac capacity is accentuated in heat‐acclimated rats [Horowitz et al., 1985]. The significant higher incidence of cardiomyopathy observed under RF‐EMR exposure further supports the notion that the heart was highly stressed (note that the overall incidence in schwannoma was not increased). The statistically significant positive trend of the malignant schwannoma incidence, with a small number of cases already observed in the 1.5 and 3 W/kg groups at both GSM and IS‐95 RF‐EMR, would indicate that already chronically mild exposure pattern‐induced temperature fluctuations might lead to adverse effects in rats.

In the 14‐week interim evaluation, DNA damage in the hippocampus was observed in comet assay data of IS‐95 RF‐EMR‐exposed male rats. At this time point, the average body mass of the rats was higher than 400g and exposure pattern‐induced SC body temperature fluctuations of more than 1°C likely happened twice a day in the 6 W/kg exposure group. Long‐lasting DNA damage in the brain of Wistar rats has been observed earlier in chronically stressed rats [Consiglio et al., 2010]. However, it is unclear whether these results can be transferred to thermoregulatory stressed situations or not. The finding of a significant upregulation of *Hsf1* along with behavioral changes in RF‐EMR‐exposed freely moving SD rats in another study suggests that SD rats are under stress if exposed with 4 W/kg for more than 6 h per day [Ohtani et al., 2016]. DNA damage was also reported in the 14‐week interim evaluation of male (frontal cortex) and female (leukocytes) mice. However, at least for the frontal cortex data of male mice, an artifact introduced by the extraordinary low value of % tail DNA in the shared sham‐exposed group cannot be ruled out [Vijayalaxmi et al., 2020] (cf. supplemental Fig. 4C in Smith‐Roe et al. [2019]).

### NTP Results Have Still to be Confirmed

An elevated incidence of schwannoma in the heart of RF‐EMR‐exposed male SD rats was also reported recently in another long‐term rodent study [Falcioni et al., 2018]. In that study, a pairwise comparison between the highest exposure (0.1 W/kg) and sham exposure (0 W/kg) groups was statistically significant, whereas the effect size was fourfold to fivefold lower than reported in National Toxicology Program (NTP) [2018a] for highly exposed SD rats. Notably, in Falcioni et al. [2018] the number of animals per exposure group was twofold to fivefold higher than in National Toxicology Program (NTP). If Falcioni et al. [2018] detected the same effect, which was observed by National Toxicology Program (NTP) [2018a], this would not be compatible with the hypothesis of thermoregulatory stress as mediating factor because in Falcioni et al. [2018] the applied wbSAR was 15–60 times lower.

**Figure 3:**
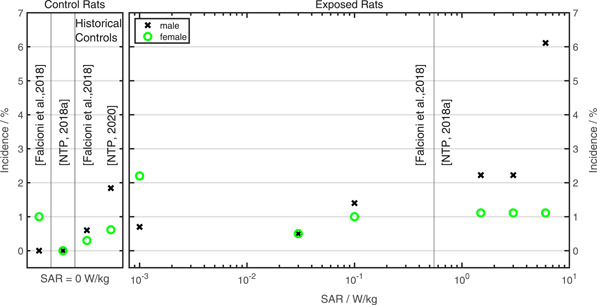
Compilation of reported incidence of schwannoma in the heart of Sprague–Dawley rats. Data taken from National Toxicology Program (NTP) [2018a] and Falcioni et al. [2018]. To obtain similar group size, GSM and IS‐95 data of National Toxicology Program (NTP) [2018a] were averaged (NTP: 2 × 90 = 180, [Falcioni et al., 2018]: approx. 200 in the maximum exposure group). Historical control data are taken from Falcioni et al. [2018] and National Toxicology Program (NTP) [2020].

In Figure 3, the incidence of schwannoma in the heart of SD rats reported in Falcioni et al. [2018] and National Toxicology Program (NTP) [2018a], as well as historical control data given in Falcioni et al. [2018] and National Toxicology Program (NTP) [2020], are compiled. In male rats the incidence strongly increases at the highest wbSAR level used by National Toxicology Program (NTP) [2018a], whereas in female SD rats no clear exposure‐related response is visible. Instead, the incidence in exposed female rats is close to what can be expected as spontaneous background rate. To our knowledge, there is no indication in the literature that the spontaneous background rate of schwannoma in the heart of rats is higher in females than in males. In light of this overall view, the single statistically significant finding in male SD rats at 0.1 W/kg [Falcioni et al., 2018] is within expectable fluctuations of the spontaneous background rate for the given group size of approximately 200 animals. Therefore, this finding is not considered as sufficiently reliable to confirm the NTP results at low wbSAR as stated by Falcioni et al. [2018] or to falsify the hypothesis of chronic thermoregulatory stress as mediating factor in the NTP study.

If the findings of the NTP study have been caused by RF‐EMR exposure, it cannot be ruled out that excessive thermoregulatory stress might be a mediating factor for the observed effects rather than a thus‐far unknown nonthermal mechanism. There is strong evidence that the overall temperature fluctuations of male rats were higher than in the other animal groups and exceeded normal physiological range. This could explain why the highest effect size was observed in exposed male rats. If the statistically significant positive trend of malignant schwannoma incidence in the heart of male rats has been caused by the body core temperature fluctuation, the results question whether a temperature increase threshold alone is applicable to account for health effects of chronic exposure situations that lead to a permanently varied active thermoregulatory response, at least in rats. To our knowledge, most research that has addressed effects of acute hyperthermia and data on effects of chronic thermoregulatory stress induced by ON/OFF switching of RF‐EMR exposure is sparse. Here, further research is necessary.

In contrast to certain chemical agents, the search for health‐related effects of chronic RF‐EMR whole‐body exposure (“hazard identification” as a goal in toxicological studies) cannot be performed in isolation from thermal effects, as the exposure is always accompanied by a certain amount of heat production which, depending on applied power, can significantly alter the living condition of the animals. This might be analogous, in part (as long as only very small body temperature elevations occur), to the effect of housing animals in different temperatures, which can lead to alterations in metabolism, cardiovascular function, respiration, immunologic function, and even cancer outcome in rodents [Hylander and Repasky, 2016]. Therefore, in future studies, it is essential to record all parameters that indicate thermally mediated effects of RF‐EMR exposure [Bundesamt für Strahlenschutz (BfS), 2019].
